# The role of WNK in modulation of KCl cotransport activity in red cells from normal individuals and patients with sickle cell anaemia

**DOI:** 10.1007/s00424-019-02327-7

**Published:** 2019-11-15

**Authors:** David C.-Y. Lu, Anke Hannemann, Rasiqh Wadud, David C. Rees, John N. Brewin, Philip S. Low, John S. Gibson

**Affiliations:** 1 Department of Veterinary Medicine, Madingley Road, Cambridge, CB3 0ES UK; 2 Department of Paediatric Haematology, King’s College Hospital, London, SE5 9RS UK; 3 Department of Chemistry, Purdue University, West Lafayette, IN 47907 USA

**Keywords:** KCl cotransport, Sickle cells, Phosphorylation, WNK, SPAK/OSR1

## Abstract

Abnormal activity of red cell KCl cotransport (KCC) is involved in pathogenesis of sickle cell anaemia (SCA). KCC-mediated solute loss causes shrinkage, concentrates HbS, and promotes HbS polymerisation. Red cell KCC also responds to various stimuli including pH, volume, urea, and oxygen tension, and regulation involves protein phosphorylation. The main aim of this study was to investigate the role of the WNK/SPAK/OSR1 pathway in sickle cells. The pan WNK inhibitor WNK463 stimulated KCC with an EC_50_ of 10.9 ± 1.1 nM and 7.9 ± 1.2 nM in sickle and normal red cells, respectively. SPAK/OSR1 inhibitors had little effect. The action of WNK463 was not additive with other kinase inhibitors (staurosporine and *N*-ethylmaleimide). Its effects were largely abrogated by pre-treatment with the phosphatase inhibitor calyculin A. WNK463 also reduced the effects of physiological KCC stimuli (pH, volume, urea) and abolished any response of KCC to changes in oxygen tension. Finally, although protein kinases have been implicated in regulation of phosphatidylserine exposure, WNK463 had no effect. Findings indicate a predominant role for WNKs in control of KCC in sickle cells but an apparent absence of downstream involvement of SPAK/OSR1. A more complete understanding of the mechanisms will inform pathogenesis whilst manipulation of WNK activity represents a potential therapeutic approach.

## Introduction

The family of cation-chloride cotransporters (CCCs) comprise the Na^+^-Cl^-^, the Na^+^-K^+^-Cl^-^, and the K^+^-Cl^-^ cotransporters (NCC, NKCCs, and KCCs). They have been identified in many tissues – notably red cells, epithelia, and neurons – in which they contribute extensively to ion and water homeostasis, both cellular and transepithelial [23]. Many of these transporters were functionally identified in the late 1970s/early 1980s as Cl^-^-dependent cation fluxes, with red cells and Ehrlich ascites tumour cells constituting pivotal model tissues [18, 28, 32, 38]. Their molecular identities were subsequently established a decade or so later [24, 46, 57]. There are two NKCC isoforms – NKCC1 is ubiquitous whilst NKCC2 is confined to the kidney – which are encoded by two genes, *SLC12A2* and *SLC12A1*, respectively. In addition, there are four KCC isoforms, encoded by *SLC12A4-7* – of which *SLC12A5* (KCC2) is found only in neurons [23]. The sole NCC isoform, *SLC12A3*, is also found in the kidney [24].

Usually, NCC and NKCCs mediate net movement of ions into cells, whilst KCCs move ions outwards. In red cells, CCCs are associated physiologically with volume regulatory processes, with NKCC involved in ion accumulation and swelling in response to shrinkage (regulatory volume increase or RVI) and KCC in ion loss and shrinkage following swelling (regulatory volume decrease, RVD) (reviewed by [9]). Physiological RVI and RVD responses, however, are not present in mature red cells from humans, although they may participate in volume regulation during erythropoiesis [21, 30]. Besides volume, red cell CCCs also respond to a number of other stimulus modalities including pH, urea, and oxygen tension [4, 25, 37, 43]. These other stimuli may represent more important modulators of KCC activity than that of volume. In addition, various stimuli, like swelling and shrinkage or oxygenation and deoxygenation, often have opposite effects on the activities of red cell NKCC and KCC [44], and these systems are often reciprocally coordinated.

In human red cells, the major significance of KCC is probably pathological in patients with sickle cell anaemia (SCA, HbSS genotype). In sickle cells, a single mutation results in the replacement of normal adult HbA with HbS. The substitution of glutamic acid with valine at position 6 of the Hb β chain allows HbS to polymerise upon deoxygenation – the initial event in the pathogenesis of SCA [5]. In patients’ red cells, over activity and also abnormal regulation of KCC contribute to excessive solute loss, with osmotically obliged water following [4, 6, 26, 35]. The ensuing shrinkage is important because increased concentration of HbS ([HbS]) markedly encourages the probability of HbS polymerisation and sickling, since the lag time to polymerisation of HbS upon deoxygenation is inversely proportional to a very high power of its concentration ([HbS]^-15-30^ is often quoted [19]). Numerous damaging sequelae follow, including altered rheology, increased fragility, intravascular haemolysis, scavenging of nitric oxide, increased red cell stickiness, thrombus formation and microvascular occlusion, and result in the plethora of clinical signs seen in SCA patients [48, 53]. Solute loss is probably a very early event in the pathogenesis of the disease following HbS polymerisation. Considerable effort has therefore been expended on understanding the underlying mechanisms and in the design of potential pharmacological inhibitors [29].

It was apparent some 30 years ago that protein phosphorylation was a key component in regulation of KCC activity, in both normal and sickle red cells from humans and across vertebrate species [10, 22, 33, 34]. Net dephosphorylation of the transporter, or a regulatory protein, was associated with higher KCC activity and net phosphorylation with reduced activity [10]. Notwithstanding, most work has been carried out using more or less specific pharmacological inhibitors (staurosporine, genistein, *N*-ethylmaleimide, calyculin A), and the identity of the specific enzymes involved remains unclear [10, 54].

An important breakthrough came when it was found that some cases of hypertension were caused by mutations in the WNK kinases [56]. It was then shown that some CCCs were regulated by two Ste20 group kinases, the oxidative stress response kinase 1 (OSR1) and the SPS1-related proline/alanine-rich kinase (SPAK or STK39) [16, 17, 47]. Later, from work mainly on epithelia, notably the kidney, it was found that the “with no lysine (K)” kinases (WNKs) both stimulated NKCC and inhibited KCC in a coordinate way, often working via downstream activation of SPAK/OSR1 [1, 14, 36]. In red cells, the situation remained unclear until more recently two papers have also revealed a role for WNKs in control of both KCC and NKCC. Working principally with the HEK293 cell line, but also with human red cells [50], showed that WNK1 inhibition played a role in stimulation of red cell KCC by swelling. Latterly, Low’s group has used transgenic mice to identify an excitatory role for WNK1 for OSR1 and, in regulation of the coordinate transporter, NKCC, upon deoxygenation [63].

Nevertheless, although in other tissues WNKs have been shown to modulate KCC activity, their role in mediating many of the stimuli affecting red cell KCC activity and their function in sickle cells remain poorly studied. In this paper, we used a the pan WNK inhibitor, WNK463, to assess the role of WNKs in regulation of KCC in red cells, mainly from SCA patients but also from normal individuals (HbAA genotype), assessing its interaction with less specific pharmacological modulators of protein phosphorylation (staurosporine, NEM and calyculin A) and with the more physiologically important stimuli (pH, volume, urea, and oxygen). Results represent the first demonstration for a pre-eminent role for WNKs in modulation of KCC activity in sickle cells, suggesting a potential key target for chemotherapeutic modulation. By contrast, pharmacological results suggest that participation of the downstream kinases SPAK/OSR1 in regulation of KCC activity was lacking.

## Materials and methods

### Materials

All chemicals and inhibitors came from Sigma-Aldrich (Poole, Dorset, UK) unless otherwise stated. WNK463 came from AdooQ Bioscience (Irvine, CA, USA), STOCK2S-26016 from Tocris Bioscience (Bristol, UK), and HK01 from ChemBridge Corporation (San Diego, CA, USA). ^86^Rb^+^ came from PerkinElmer (Beaconsfield, Bucks., UK). Nitrogen was from BOC Ltd (Guildford, Surrey, UK).

### Blood samples

Consented samples were acquired with ethical approval from patients with sickle cell anaemia (SCA, genotype HbSS – termed HbSS cells) or normal individuals (genotype HbAA – termed HbAA cells) using EDTA as anticoagulant (REC reference number 16/LO/1309). Occasionally routine discarded blood samples left over from clinical assays were also used. All samples were obtained from the Sickle Cell Clinic at King’s College Hospital and were anonymised. Samples were refrigerated until used, within 2 days. Whole blood was then washed in Cl^-^-free saline (N-MBS, see below) to remove plasma, buffy coat, and also Cl^-^, and red cells are stored on ice until required.

### Salines and inhibitors

Nitrate-containing MOPS-buffered saline (N-MBS) comprised (in mM) NaNO_3_ 145, MOPS 10, glucose 5, and pH 7.4 at 37 ^°^C. Cl^-^-containing MBS (Cl-MBS) had similar composition but with NaCl replacing NaNO_3_. Wash solution (W-MBS) was isotonic MgCl_2_ solution: MgCl_2_ 107, MOPS 10, and pH 7.4 at 0 ^°^C.

### Tonometry

KCC activity in human red cells is O_2_-sensitive [26]. It was therefore important to regulate O_2_ tension during incubation. Cells were gently rotated at 37 ^°^C in Eschweiler tonometers, coupled to a Wösthoff gas mixing pump to set the O_2_ tension at the requisite level from 150 mmHg oxygen to 0 by mixing pre-warmed and humidified air and N_2_. Typically, cells were placed in the tonometers at tenfold the haematocrit (Hct) needed for transport assay and equilibrated at the requisite O_2_ tension. They were then diluted tenfold into test tubes, also pre-equilibrated at the required O_2_ level. Tubes were also gassed during incubation, but not bubbled (to prevent red cell lysis). Humidified gas is necessary to prevent dehydration of the samples and to prevent condensation; all glassware and tubing were submerged and kept at 37 ^°^C.

### Measurement of KCC activity

^86^Rb^+^ was used as a K^+^ congener. After dilution of the red cell samples into the test tubes, the influx was started by addition of ^86^Rb^+^ (final activity about 0.05 MBq.ml^-1^) to warm (37 ^°^C) cell suspensions. ^86^Rb^+^ was added in a solution of 150 mM KNO_3_ added at a 1 in 20 dilution to give a final extracellular [K^+^] of 7.5 mM. The duration of uptake here was 10 min, control experiments have established that uptake is linear over this time period, and determinations were usually carried out in triplicates. Uptake was stopped by diluting aliquots of the cell suspension into ice-cold W-MBS. Unincorporated ^86^Rb^+^ was removed by centrifugation (10 s at 15,000 g), aspiration of supernatant, and addition of further wash solution (4 washes and 5 spins in total). After each centrifugation step, cells were resuspended by gentle vortexing. Following the final wash, the cell pellet was lysed with Triton X-100 (0.1%) and protein (mainly haemoglobin in the case of red blood cells) precipitated with trichloroacetic acid (TCA, 5%). A final centrifugation step was used to separate off the clear, colourless supernatant before counting. Activity was measured as Čerenkov radiation by liquid scintillation (Packard Tri-carb 2800TR). The test tubes contained transport inhibitors in Cl-MBS or N-MBS as required. KCC activity was calculated as the Cl^-^-dependent K^+^ uptake and given as mmol K^+^(l cells.h)^-1^. Ouabain (100 μM) and bumetanide (10 μM) were present during all influx assays to inhibit K^+^ uptake via the Na^+^/K^+^ pump and Na^+^-K^+^-2Cl^-^ cotransporter (NKCC), respectively.

### Inhibitor studies

**Fig. 1:**
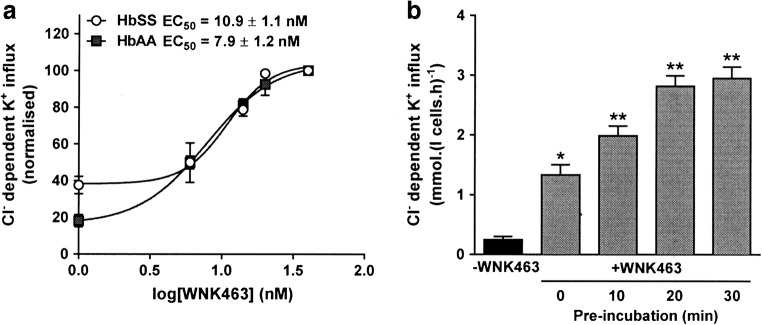
Effect of WNK463 on KCl cotransport (KCC) activity in red cells from normal individuals (HbAA) and patients with sickle cell anaemia (SCA). Red cells from patients homozygous for SCA (20% haematocrit, Hct) or healthy individuals (40% Hct) were pre-incubated in N-MBS for 30 min at 37 °C in air in the presence of 0–40 nM WNK463, unless stated otherwise. They were then equilibrated in Eschweiler tonometers for 20 min in air (150 mmHg O_2_) in the continued presence of WNK463, after which aliquots were diluted tenfold into flux tubes. KCC activity was measured as Cl^-^-dependent K^+^-influx for 10 min at an extracellular [K^+^] of 7.5 mM. KCC activity is given in mmol.(l cells.h)^-1^. Ouabain (100 μM) and bumetanide (10 μM) were present in all experiments. **a** Effect of 0–40 nM WNK463 on KCC activity. KCC activity was normalised to that at 40 nM WNK463 and EC_50_ calculated using nonlinear regression. **b** Effect of duration of pre-incubation with 40 nM WNK463 on KCC activity in HbSS cells. Symbols represent means ± SEM, n = 3. * p < 0.05, ** p < 0.01 compared to red cells incubated in the absence of WNK463

For most experiments, red cells were pre-incubated with inhibitors for 30 min at 20% haematocrit (Hct) at pH 7.4 under isotonic conditions (290 mOsm.kg^-1^) at 37 °C. They were then equilibrated in tonometers either fully oxygenated or at the required oxygen tension (Fig. 6) for a further 20 min. Red cells were then diluted tenfold into test tubes at the required pH (pH 7 or 7.4), tonicity (10% shrunken through addition of hypertonic sucrose or 10% swollen through addition of water) or with added urea (500 mM), and KCC activity measured. For the experiments in Fig. 1b, pre-incubation with WNK463 was varied from 0 up to 30 min before equilibrating in tonometers in the presence WNK463 for 20 min and subsequent measuring of KCC activity. For combinations of WNK463 and staurosporine, NEM, or calyculin A, red cells were exposed sequentially to each inhibitor (or DMSO solvent) for 30 min.

### Phosphatidylserine exposure

Phosphatidylserine exposure was measured by FACS using FITC-labelled lactadherin (see [12] for details).

### Statistics

Data are given as means ± S.E.M. for samples from n different individuals. Comparisons with and without inhibitors were carried out in paired samples, and statistical analysis was made using student’s t test. A value of p < 0.05 was taken as significant.

## Results

### The effect of WNK463 on KCl cotransport in human red cells

In the first series of experiment, the effect of the pan WNK inhibitor WNK463 was examined in red cells from both normal individuals (termed HbAA cells) and patients with SCA (termed HbSS cells). Initially, its concentration dependence was investigated. WNK463 increased KCC activity in both HbAA and HbSS cells, with an EC_50_ of 7.9 ± 1.2 nM and 10.9 ± 1.6 nM, respectively (Fig. 1a). The time course of the effect of WNK463 was also determined. After equilibrating in the presence of WNK463 in fully oxygenated conditions for 20 min, the inhibitor significantly increased KCC activity by about fivefold (Fig. 1b). With additional periods of pre-incubation prior to oxygen tension equilibration, the stimulatory effect of WNK463 increased, becoming maximal after 20 min pre-incubation. On the basis of these preliminary experiments, a concentration of 40 nM WNK463 and a pre-incubation time of 30 min were chosen for subsequent work. In the absence of Cl^-^, in N-MBS, K^+^ influxes were 0.65 ± 0.10 and 0.70 ± 0.20 mmol.(l cells.h)^-1^ in normal and sickle red cells in the absence of WNK463 and 0.74 ± 0.10 and 0.78 ± 0.15 in its presence (40 nM), means ± S.E.M., n = 3 (all N.S.), confirming that the major effect of WNK463 was mediated via KCC activity. The following is largely restricted to work in red cells from SCA patients, but similar findings were obtained with those from normal HbAA individuals and are given in brief.

### The effect of combinations of WNK463 and staurosporine, N-ethylmaleimide (NEM), and calyculin A in HbSS cells and HbAA cells

**Fig. 2:**
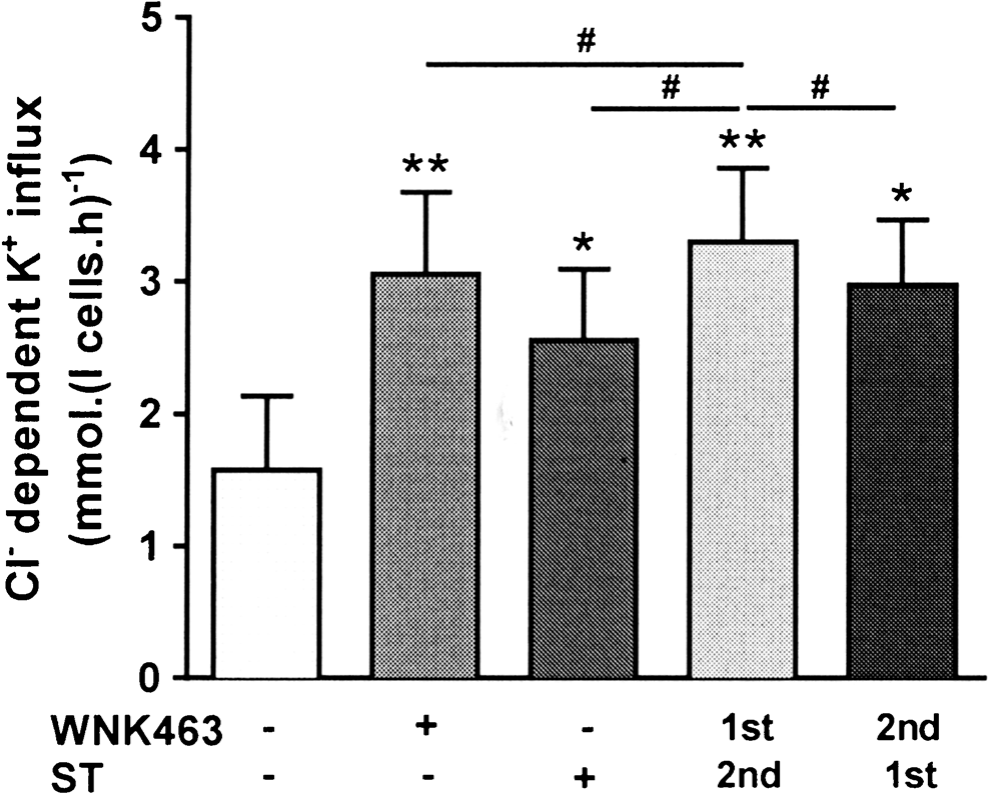
Effect of staurosporine and WNK463 on KCC activity in red cells from patients with SCA. Red cells (20% Hct) were pre-incubated in N-MBS sequentially for two periods of 30 min in the presence of vehicle (DMSO) or drug (WNK463 40 nM or staurosporine 100 μM), as indicated. They were then equilibrated in Eschweiler tonometers for 20 min at 150 mmHg in the continued presence of WNK463 and/or staurosporine and KCC activity measured as described in the legend to Fig. 1. Histograms represent means ± SEM, n = 4. * p < 0.05, ** p < 0.01 compared to red cells incubated in the absence of WNK463; ^#^ p < 0.05 between groups as indicated

Staurosporine (100 μM) represents one of the main protein kinase (PK) inhibitors used to stimulate KCC activity in red cells [10]. Its effects were compared with those of WNK463 (Fig. 2). When incubated with each PK inhibitor alone, the stimulatory effects of WNK463 and staurosporine were similar, albeit slightly greater for WNK463. Sequential application of the two inhibitors also gave similar levels of activity although addition of WNK463 before staurosporine appeared to slightly increase KCC activity further compared to either inhibitor alone. However, the actual increase of KCC activity was only 10.6 ± 5.1% compared to WNK463 alone, suggesting a similar target kinase for both reagents.

**Fig. 3:**
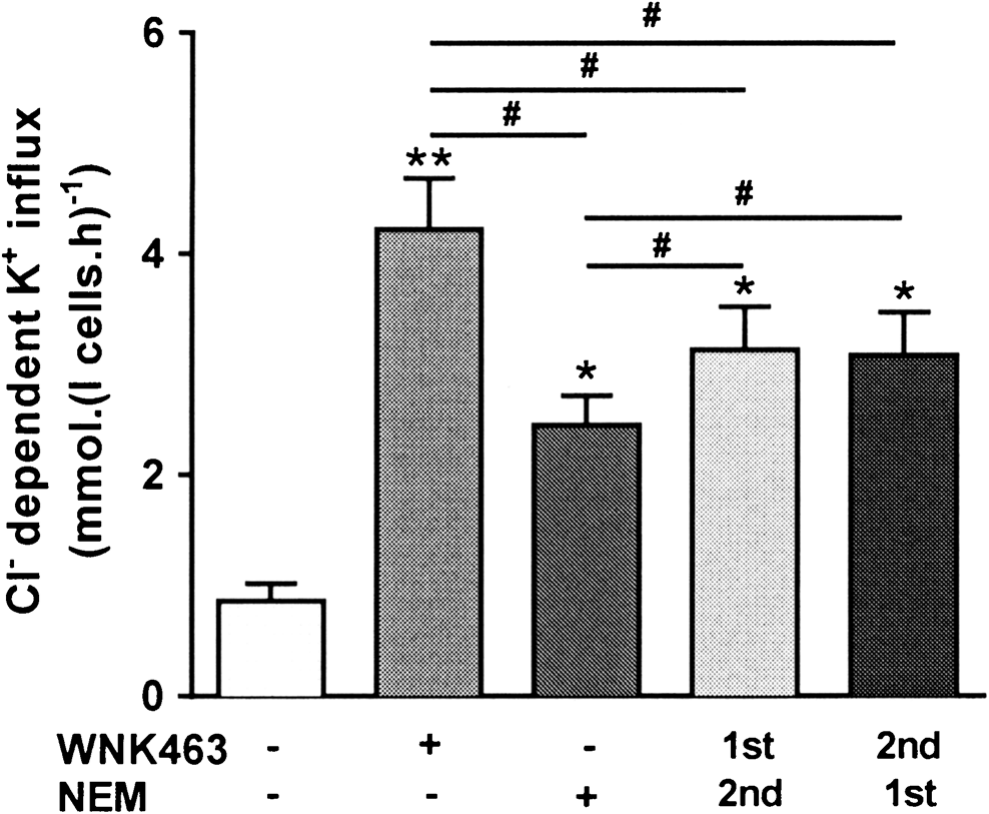
Effect of *N*-ethylmaleimide (NEM) and WNK463 on KCC activity in red cells from patients with SCA. Red cells (20% Hct) were pre-incubated in N-MBS sequentially for two periods of 30 min in the presence of vehicle (DMSO) or drug (WNK463 40 nM or NEM 1 mM), as indicated. They were then equilibrated in Eschweiler tonometers for 20 min at 150 mmHg in the continued presence of WNK463 and/or NEM and KCC activity measured as described in the legend to Fig. 1. Histograms represent means ± SEM, n = 4. * p < 0.05, ** p < 0.01 compared to red cells incubated in the absence of WNK463; ^#^ p < 0.05 between groups as indicated

A second putative PK inhibitor used to stimulate KCC activity in red cells has been the thiol-reacting reagent *N*-ethylmaleimide (NEM; 100 μM) [20, 38]. When compared with WNK463, NEM alone or in combination with WNK463, NEM gave significantly lower levels of KCC activity compared to WNK463 alone, whether applied prior to or after WNK463 (Fig. 3). Again, there was no indication of additive effects of the two reagents. As NEM/WNK463 combinations always reduced KCC activity below that of WNK463 alone, it suggested that effects other than PK inhibition were present.

**Fig. 4:**
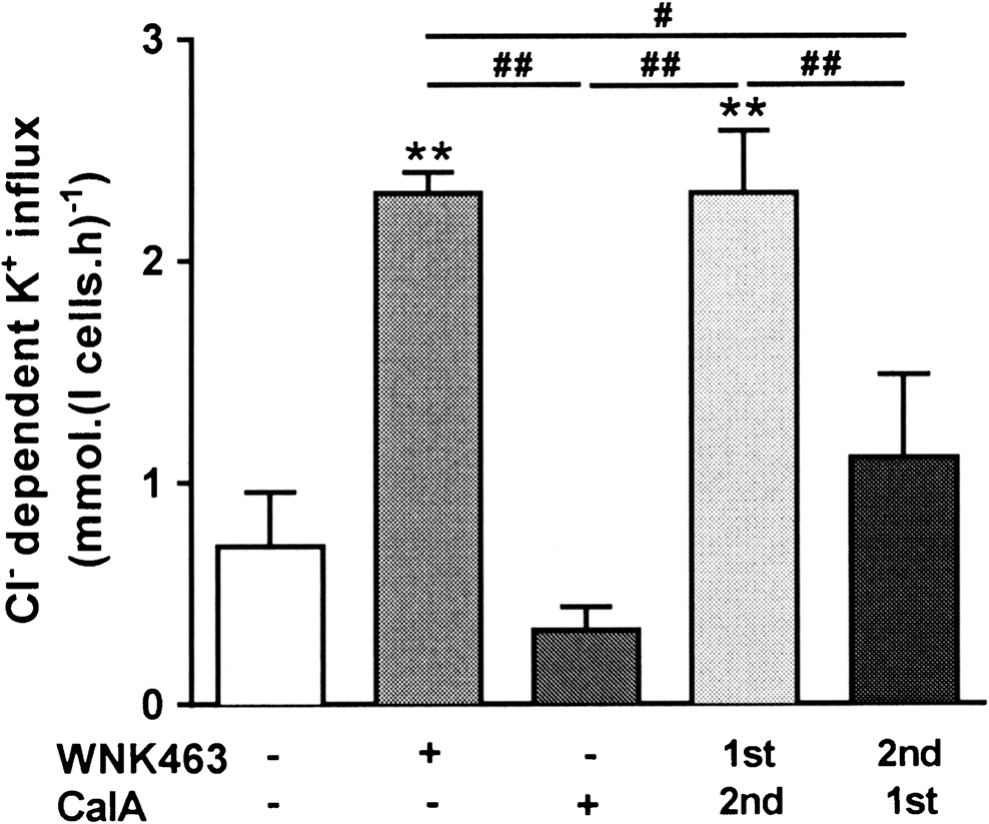
Effect of calyculin A and WNK463 on KCC activity in red cells from patients with SCA. Red cells (20% Hct) were pre-incubated in N-MBS sequentially for two periods of 30 min in the presence of vehicle (DMSO) or drug (WNK463 40 nM or calyculin A 100 nM), as indicated. They were then equilibrated in Eschweiler tonometers for 20 min at 150 mmHg in the continued presence of WNK463 and/or calyculin A and KCC activity measured as described in the legend to Fig. 1. Histograms represent means ± SEM, n = 6. ** p < 0.01 compared to red cells incubated in the absence of WNK463; ^#^ p < 0.05, ^##^ p < 0.01 between groups as indicated.

The protein phosphatase inhibitor calyculin A (100 nM) has been shown to inhibit KCC activity in red cells and to prevent subsequent stimulation by PK inhibitors following pre-incubation with calyculin A [52]. These observations suggest that dephosphorylation of the regulatory site controlling KCC activity involves a calyculin A-sensitive phosphatase PP1 and PP2a [2, 3]. As observed previously, calyculin A on its own inhibited KCC activity below that in control cells (Fig. 4). Following WNK463 addition, however, calyculin A had minimal effect. When added prior to WNK463, calyculin A greatly reduced the stimulatory effect of subsequent addition of WNK43. These findings are consistent with the WNK phosphoresidue target also being dephosphorylated by a calyculin A-sensitive phosphatase.

Similar findings were obtained with red cells from normal individuals (HbAA). Control K^+^ influxes in the absence of protein kinase/phosphatase inhibitors were 0.2 to 0.3 mmol.(l cells.h)^-1^. In the presence of staurosporine (100 μM) and NEM (1 mM), these increased to 3.41 ± 0.86 and 3.01 ± 0.60 mmol.(l cells.h)^-1^, respectively. When treated with combination of staurosporine or NEM and WNK463 (40 nM), influxes were 3.5 ± 0.5 and 3.55 ± 0.80 mmol.(l cells.h)^-1^ (means ± S.E.M, n = 3; N.S. cf staurosporine and NEM alone), respectively – showing that the action of WNK463 and staurosporine/NEM was not additive. With calyculin A (100 nM), influxes were reduced from 0.30 ± 0.07 to 0.20 ± 0.06 mmol.(l cells.h)^-1^ increasing to 3.40 ± 0.82 with WNK463 alone, and in combination with WNK463 after calyculin A, they were 0.43 ± 0.16 mmol.(l cells.h)^-1^, showing that pre-treatment of red cells with the protein phosphatase inhibitor calyculin A prevented KCC in normal red cells from responding to WNK463.

### The effect of combinations of WNK463 and physiological stimuli modulating KCC activity in HbSS cells

**Fig. 5:**
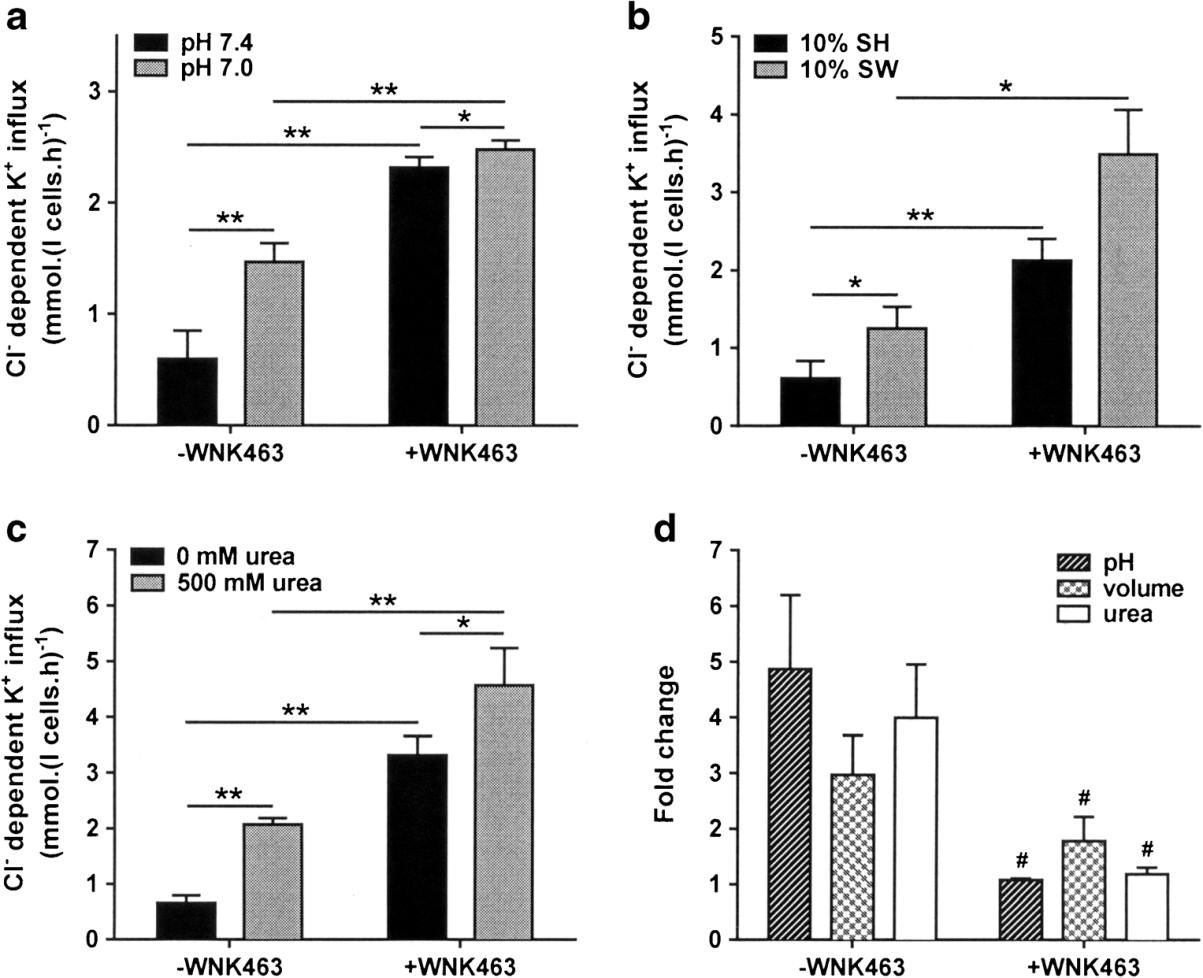
Effect of WNK463 on physiological stimuli of KCC in red cells from patients with SCA. Red cells (20% Hct) were pre-incubated in N-MBS in the presence or absence of WNK463 (40 nM) at 37 °C in air. They were then equilibrated in Eschweiler tonometers for 20 min at 150 mmHg, after which aliquots were diluted tenfold into flux tubes containing buffers varying in pH, osmolarity, or urea content, all in the continued presence or absence of WNK463, and KCC activity was measured as described in the legend to Fig. 1. **a** Effect of WNK463 on pH-dependent KCC activity, n = 6. **b** Effect of WNK463 on KCC activity in 10% shrunken (SH) or 10% swollen (SW) red cells, n = 5. **c** Effect of WNK463 on urea-induced KCC activity, n = 6. **d** Impact of WNK463 on pH, volume, and urea-stimulated KCC activity. Histograms represent means ± SEM of n individual samples. * p < 0.05, ** p < 0.01, *** p < 0.001 compared to indicated condition, ^#^ p < 0.05 compared to fold change in absence of WNK463

The effect of changes in pH and volume change and also incubation with high concentrations of urea was compared in control HbSS cells and following pre-incubation with WNK463. All three stimuli significantly elevated KCC activity – as shown previously [25] – but none stimulated activity to the extent achieved by WNK463 alone (Fig. 5a–c). Notwithstanding all three were still able to increase KCC activity following pre-incubation with WNK463, although the fold changes in activity were considerably reduced compared with those in cells not pre-incubated with WNK463 (Fig. 5d). These findings may indicate that whilst these other stimuli may act mainly through WNK inhibition, their effect must also be mediated via some other mechanism, as suggested for the coordinate transport NKCC in HEK293 cells [31]. Conversely, it may be that pre-incubation was insufficient to completely abrogate WNK activity. A similar pattern was also found in normal HbAA red cells.

**Fig. 6:**
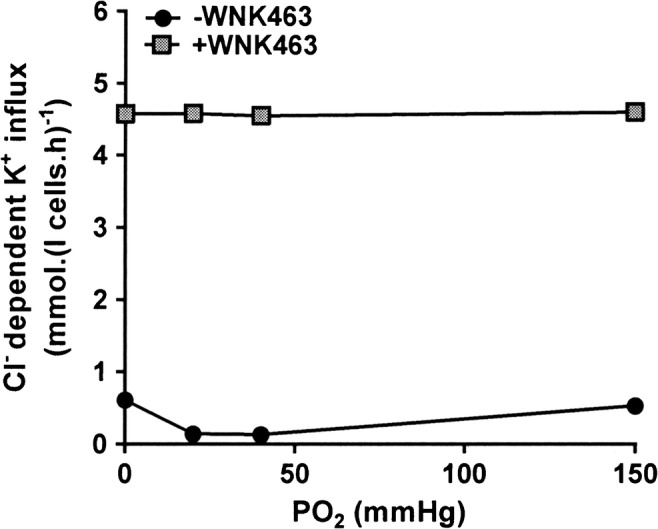
Effect WNK463 on oxygen-dependent KCC activity in red cells from patients with SCA. Red cells (20% Hct) were pre-incubated in N-MBS in the presence or absence of WNK463 (40 nM) at 37 °C in air. They were then equilibrated in Eschweiler tonometers for 20 min at 0–150 mmHg, after which aliquots were diluted tenfold into flux tubes, all in the continued presence or absence of WNK463, and KCC activity was measured as described in the legend to Fig. 1. Symbols represent means ± SEM (error bars are smaller than symbols), n = 6

In the case of red cell NKCC, WNK1 appears to be responsible for increased activity following deoxygenation [63]. KCC activity is also oxygen dependent, although in a reciprocal fashion being activated by oxygenation rather than deoxygenation. In red cells from normal individuals, KCC is maximally active under conditions of full oxygenation, with activity declining as oxygen tension is lowered such that the transporter is inactive when cells are fully deoxygenated [26]. In sickle cells, KCC activity has an abnormal oxygen dependence, with highest activity in fully oxygenated and fully deoxygenated cells with a nadir at about the *P*O_2_ required for half maximal saturation of Hb with oxygen [26]. This abnormal oxygen dependence was confirmed here (Fig. 6). The interaction of WNK and oxygen tension was also investigated. When pre-incubated with WNK463, KCC activity was maximally stimulated and became insensitive to changes in oxygen tension. These findings suggest a role for WNK in mediating the oxygen sensitivity of KCC, as well as for that of NKCC.

### The effect of inhibitors of SPAK/OSR1

**Fig. 7:**
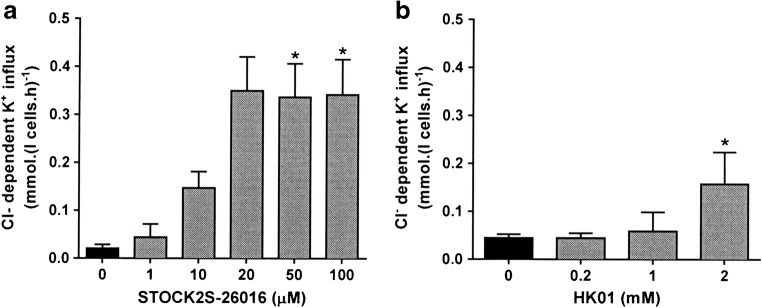
Effect of SPAK/OSR1 inhibitors on KCC activity in HbAA RBCs. Red cells (40% Hct) were pre-incubated in N-MBS in the presence of 0–100 μM STOCK2S-26016 or 0–2 mM HK01 at 37 °C in air. They were then equilibrated in Eschweiler tonometers for 20 min at 0–150 mmHg, after which KCC activity was measured as described in the legend to Fig. 1. **a** Effect of STOCK2S-26016 on KCC activity in HbAA cells, n = 3. **b** Effect of HK01 on KCC activity in HbAA cells, n = 3. Histograms represent means ± SEM of n individual samples. * p < 0.05

In many cases, SPAK/OSR1 are implicated as a downstream target of WNKs. Following phosphorylation by WNKs, SPAK/OSR1 then carry out phosphorylation of the relevant CCC. The effects of several SPAK/OSR1 inhibitors (STOCK2S-26016, closantel, and rafoxanide) as well as of HK01, an inhibitor of MO25, a scaffolding protein that increases SPAK/OSR1 activity > 100-fold, were investigated. None of these inhibitors, however, gave comparable effects to that of WNK463. STOCK2S-26016 and HK01 did stimulate a Cl^-^-dependent K^+^ influx, but effects were minimal (Fig. 7a and b for healthy HbAA red cells) whilst 40 nM WNK463 increased it about tenfold, from 0.35 ± 0.04 to 3.1 ± 0.2 mmol.(l cells.h)^-1^ (n = 24). In comparison, in red cells from HbSS patients Cl^-^-dependent K^+^-influx increased from 0.48 ± 0.2 to 1.31 ± 0.3 mmol.(l cells.h)^-1^ in the presence of HK01 (p < 0.021; n = 3) and from 0.70 ± 0.10 to 3.3 ± 0.2 mmol.(l cells.h)^-1^ (n = 3) in the presence of 40 nM WNK463. Both closantel and rafoxanide also increased K^+^ influx, but in this case, transport was not Cl^-^-dependent suggesting a nonspecific increase in membrane permeability rather than stimulation of KCC (data not shown). These findings are evidence against a major role for SPAK/OSR1 in the phosphorylation pathway modulating red cell KCC activity.

### The effect of WNK463 on phosphatidylserine exposure

As well as solute loss through KCC activity and other pathways, phosphatidylserine (PS) exposure is also implicated in the pathogenesis of SCA as it is prothrombotic and contributes to both anaemia and ischaemia. It has also been shown to be affected by protein phosphorylation and is inhibited by some protein kinase inhibitors, notably PKC inhibitors [55]. It was therefore pertinent to determine whether WNK inhibition had any effect on PS externalisation. There was no apparent effect of WNK463, however, either in control HbSS cells or those loaded with Ca^2+^ (1 μM) using the ionophore bromo-A23187 (6 μM). For example, in Ca^2+^-loaded cells, the percentage of cells positive for externalised PS was 47.2 ± 6.0 % in the absence of WNK463 and 43.2 ± 7.2 % in its presence (both means ± S.E.M., n = 4, N.S.). Similar findings were found in red cells from normal individuals. A similar lack of effect was found in red cells from normal individuals, in which, in the absence of ionophore, PS exposure was 0.8 ± 0.1% in controls and 0.8 ± 0.1% following treatment with WNK463.

## Discussion

Red cell KCC is sensitive to a number of stimuli including volume, pH, urea, and oxygen tension [25]. These modalities appear to affect the transporter by protein (de)phosphorylation [33, 34], with pharmacological evidence for the presence of both serine/threonine and tyrosine phosphoresidues [2, 10]. Knockout studies in mice have indicated a role for the Src and Syk tyrosine kinases [13, 41, 42], with the conjugate phosphatases identified as PP1, PP2A, or PP2B [2, 3]. More recent publications provide molecular evidence for a role for WNK1 and possibly the functionally redundant WNK substrates SPAK/OSR1 in inhibition of KCC and stimulation of coordinate cotransport, NKCC, in red cells [15, 50, 63]. This paper provides the first demonstration of a functional role for WNKs in control of KCl cotransport in red cells from patients with sickle cell anaemia (SCA).

The main red cell KCC isoform(s) remain(s) uncertain. Early studies suggested the presence of KCC1, KCC3, and KCC4 [39]. Later, KCC1 and KCC3 were found to be predominant, with KCC3 probably being the main KCC in normal human red cells [45, 51], although the main red cell isoforms in other species may vary. In addition, in sickle cells, normal expression of KCC isoforms may be disrupted [11]. Thus whilst all three isoforms were identified in red cells from SCA patients, several splice variants were present, and one (of KCC1) differed quantitatively compared to red cells from normal individuals [11]. It is not clear to what extent the presence of different forms of KCC affects the function.

The present work makes use of the pan WNK inhibitor, WNK463. This inhibitor produced a marked activation of KCC with an EC_50_ of around 10 nM, which is about the concentration reported in the literature for a specific effect on WNKs. The EC_50_ of WNK463 for different WNKs varies and is reported to be 5, 1, 6, and 9 nM for WNKs 1, 2, 3, and 4, respectively [60]. Our functional assays of KCC activity, however, are insufficiently sensitive to use these small concentration differences to identify the main red cell WNK. The EC_50_ of WNK463 is very similar to that reported in the literature for WNKs which is consistent with an action on these enzymes. An important caveat, however, is that definitive proof of the role of WNKs awaits phosphorylation studies in mature red cells or knockdown assays in nucleated red cell precursors. Nevertheless, as the abnormally high activity of KCC in red cells from sickle cell patients is known to mediate solute loss and decrease in cell volume, KCC stimulation induced by WNK463 would be expected to result in shrinkage.

Both hypertonicity and a reduction of internal [Cl^-^] have been shown to activate WNKs [49, 58, 61] leading to an inhibition of KCC activity. In addition, hypotonicity, sometimes in combination with high [K^+^], had the opposite effect, decreasing WNK phosphorylation and activating KCC [15, 62]. Here, hypotonicity, low pH, and urea were used to increase KCC activity which was increased further by treatment with WNK463. Notwithstanding, the sensitivity to these physiological stimuli was significantly reduced in the presence WNK463, consistent with the involvement of WNKs, and widening the stimuli with which these enzymes are associated.

Oxygen is another physiological regulator of KCC and NKCC in red cells. Until recently the mechanism was unknown although haemoglobin (Hb) represented the most obvious sensor [27]. Deoxyhaemoglobin has profound effects on red cell function, acting largely through its greater affinity for the cytoplasmic tail of band 3 (AE1) compared to oxyhaemoglobin and from which it displaces several proteins, including several glycolytic enzymes and ankyrin [7, 40]. In a similar way, deoxyHb was also recently found to compete with WNK1 following which its release into the cytoplasm led to OSR1 activation and subsequent NKCC1 phosphorylation and activation [63]. As NKCC and KCC are often regulated reciprocally, activation of WNK1 by deoxygenation would explain phosphorylation and inactivation of KCC. In SCA, however, KCC activity is abnormally high, and its aberrant response to deoxygenation – an increase in activity as oxygen tension falls from the *P*O_2_ of Hb to 0 mmHg – may be explained if polymerisation of HbS removes the source of deoxyHb for WNK displacement leading to decreased WNK activity. The present findings show that inhibiting WNK with WNK643 not only significantly increased KCC activity but also abrogated its oxygen dependence.

In previous work in HEK293 cell lines, a major role for WNK1 was implicated in control of KCC3 activity using RNA interference (RNAi), with less evidence for WNK2 and WNK4 [50]. In these cells, WNK1 inhibition activated volume-sensitive KCC3 activity via dephosphorylation of T991 and T1048. The same residues were dephosphorylated in hypotonically induced KCC3 activity in red cells, although no evidence was presented for which WNK was involved. Phosphorylation of KCC3 T1048 – and its equivalent in the other KCC isoforms – was later shown to be mediated by SPAK/OSR1 whilst that of T991 was not [15, 62], with neither residue being directly phosphorylated by WNK1 or WNK3. In the present study, several SPAK/OSR1 inhibitors with different mechanisms of action were tested (STOCK2S-26016, HK01, closantel, and rafoxanide), but their impact on red cell KCC activity was minimal.

This apparent paradox could be explained by several observations. When either T991 or T1048 were mutated to alanine, KCC activity increased moderately and could still be modulated by low internal [Cl^-^] or volume change. By contrast, T991A/T1048A double mutants were highly active, and the incubation medium had no further impact. Furthermore, in vitro phosphorylation experiments using purified KCC and SPAK or OSR1 showed phosphorylation of T1048 but not T991 [15, 50], whilst in an ES knockin model lacking SPAK and OSR1 activity, only T991 was phosphorylated [15]. The effect of a siRNA knockdown of WNK1 in HEK293 was less clear. It markedly reduced KCC phosphorylation, whilst knockdown of SPAK or OSR1 did not [50]. It is unclear, however, if the knockdowns of SPAK and OSR1 were simultaneous or separate. If the latter was the case, then one enzyme could have compensated for the loss of the other. Should SPAK/OSR1 only be involved in the phosphorylation of T1048 but that of both T991 and T1048 are required for a full impact on KCC regulation, inhibiting SPAK/OSR1 would be expected to have a much smaller effect than inhibiting upstream WNK. Further understanding of the pathways controlling KCC in red cells awaits identification of the phosphoresidues involved.

Other pharmacological inhibitors of protein phosphorylation also increased KCC activity. Staurosporine, which interacts with over two hundred and fifty human kinases with varying potency, showed a similar effect to WNK463, whilst a combination of both did not appear to be additive. Moreover, it has been shown to inhibit WNK1 directly in vitro in an ATP-dependent manner [59], providing a possible mechanism for its observed action on KCC activity and lack of any additive effects. NEM acts via modification of sulfhydryl group of cysteine residues and has been shown to decrease phosphorylation at an established WNK phosphorylation site in SPAK required for its activity, as well as at the T1048 equivalent in KCC2 leading to increased activity in KCC2 transfected HEK293 cells [8]. Whilst KCC2 is not present in red cells, these findings suggest a possible mechanism for KCC activation by NEM. Finally, the effect of WNK inhibition was largely abrogated by pre-treatment with calyculin A, indicating a role for protein phosphatases PP1 and PP2A in dephosphorylation of the WNK target.

In conclusion, the present findings confirm the involvement of WNK in negative regulation of KCC activity in human red cells. Whilst this is not a surprise and has been shown for several tissues, notably epithelia and neurons, there is little information on its role in red cells aside from a single report on hypotonically induced KCC activity and, to date, nothing on sickle cells. In addition, results further emphasise the role of WNKs in influencing KCC activity by important physiological modulators – volume, pH, urea, and oxygen tension.

## Abbreviations

CCC: Cation-chloride cotransporters
KCC: K^+^-Cl^-^ cotransporter
NCC: Na^+^-Cl^-^ cotransporter
NKCC: Na^+^-K^+^-2Cl^-^ cotransporter
ORS1: The oxidative stress response kinase 1 (OSR1)
RVD: Regulatory volume decrease
RVI: Regulatory volume increase
SCA: Sickle cell anaemia
SLC: Solute-linked carrier transport protein
SPAK: The SPS1-related proline/alanine-rich kinase (SPAK or STK39)
WNK: “With no lysine (K)” kinases

## References

[1] Alessi DR, Zhang J, Khanna A, Hochdorfer T, Shang Y, Kahle KT (2014). The WNK-SPAK/OSR1 pathway: master regulator of cation-chloride cotransporters. Sci Signal.

[2] Bize I, Guvenc B, Robb A, Buchbinder G, Brugnara C (1999). Serine/threonine protein phosphatases and regulation of K-Cl cotransport in human erythrocytes. Am J Physiol.

[3] Bize I, Guvenc B, Buchbinder G, Brugnara C (2000). Stimulation of human erythrocyte K-Cl cotransport and protein phosphatase type 2A by n-ethylmaleimide: role of intracellular Mg^2+^. J Membr Biol.

[4] Brugnara C, Bunn HF, Tosteson DC (1986). Regulation of erythrocyte cation and water content in sickle cell anemia. Science.

[5] Bunn HF, Forget BG (1986). Hemoglobin: molecular, genetic and clinical aspects.

[6] Canessa M, Spalvins A, Nagel RL (1986). Volume-dependent and NEM-stimulated K^+^, Cl^-^ transport is elevated in oxygenated SS, SC and CC human red cells. FEBS Lett.

[7] Chu H, McKenna MM, Krump NA, Zheng S, Mendelsohn L, Thien SL, Garrett LJ, Bodine DM, Low PS (2016). Reversible binding of hemoglobin to band 3 constitutes the molecular switch that mediates O_2_ regulation of erythrocyte properties. Blood.

[8] Conway LC, Cardarelli RA, Moore YE, Jones K, McWilliams LJ, Baker DJ, Burnham MP, Burli RW, Wang Q, Brandon NJ, Moss SJ, Deeb TZ (2017). N-ethylmaleimide increases KCC2 cotransporter activity by modulating transporter phosphorylation. J Biol Chem.

[9] Cossins AR, Gibson JS (1997). Volume-sensitive transport systems and volume homeostasis in vertebrate red blood cells. J Exp Biol.

[10] Cossins AR, Weaver YR, Lykkeboe G, Nielsen OB (1994). Role of protein phosphorylation in control of K flux pathways of trout red blood cells. Am J Physiol.

[11] Crable SC, Hammond SM, Papes R, Rettig RK, Zhou G-P, Gallagher PG, Joiner CH, Anderson KP (2005). Multiple isoforms of the KCl cotransporter are expressed in sickle and normal erythroid cells. Exp Hematol.

[12] Cytlak UM, Hannemann A, Rees DC, Gibson JS (2013). Identification of the Ca^2+^ entry pathway involved in deoxygenation-induced phosphatidylserine exposure in red blood cells from patients with sickle cell disease. Pflugers Arch - Eur J Physiol.

[13] De Franceschi L, Fumagalli L, Olivieri O, Corrocher R, Lowell CA, Berton G (1997). Deficiency of Src family kinases Fgr and Hck results in activation of erythrocyte K/Cl cotransport. J Clin Investig.

[14] de los Heros P, Kahle KT, Rinehart J, Bobadilla NA, Vazquez N, San Cristobal P, Mount DB, Lifton RP, Hebert SC, Gamba G (2006). WNK3 bypasses the tonicity requirement for K-Cl cotransporter activation via a phosphatase-dependent pathway. Proc Natl Acad Sci U S A.

[15] de los Heros P, Alessi DR, Gourlay R, Campbell DG, Deak M, Macartney TJ, Kahle KT, Zhang J (2014). The WNK-regulated SPAK-OSR1 kinases directly phosphorylate and inhibit the K^+^-Cl^-^ co-transporters. Biochem J.

[16] Delpire E, Gagnon KBE (2008). SPAK and OSR1: STE20 kinases involved in the regulation of ion homeostasis and volume control in mammalian cells. Biochem J.

[17] Dowd BFX, Forbush B (2003). PASK (proline-alanine-rich STE20-related kinase), a regulatory kinase of the Na-K-l cotransporter (NKCC1). J Biol Chem.

[18] Dunham PB, Ellory JC (1980). Chloride-activated potassium transport in human erythrocytes. Proc Natl Acad Sci U S A.

[19] Eaton JW, Hofrichter J (1987). Hemoglobin S gelation and sickle cell disease. Blood.

[20] Ellory JC, Dunham PB (1982). Logue PJ, and Stewart GW (1988) Anion-dependent cation transport in erythrocytes. Phil Trans R Soc Lond B.

[21] Ellory JC, Hall AC (1988). Human red cell volume regulation in hypotonic media. Comp Biochem Physiol.

[22] Flatman PW, Adragna NC, Lauf PK (1996). Role of protein kinases in regulating sheep erythrocyte K-Cl cotransport. Am J Physiol.

[23] Gamba G (2005). Molecular physiology and pathophysiology of electroneutral cation-chloride cotransporters. Physiol Rev.

[24] Gamba G, Saltzberg SN, Lombardi M, Miyanoshita A, Lytton J, Hediger MA, Brenner BM, Hebert SC (1993). Primary structure and functional expression of a cDNA encoding the thiazide-sensitive, electroneutral sodium-chloride cotransporter. Proc Natl Acad Sci U S A.

[25] Gibson JS, Ellory JC, Bernhardt I, Ellory JC (2003). K^+^-Cl^-^ cotransport in vertebrate red cells. Red cell membrane transport in health and disease.

[26] Gibson JS, Speake PF, Ellory JC (1998). Differential oxygen sensitivity of the K^+^-Cl^-^ cotransporter in normal and sickle human red blood cells. J Physiol.

[27] Gibson JS, Cossins AR, Ellory JC (2000). Oxygen-sensitive membrane transporters in vertebrate red cells. J Exp Biol.

[28] Gibson JS, Ellory JC, Adragna NC, Lauf PK, Delpire FJA-LE (2009). Pathophysiology of the K^+^-Cl^-^ cotransporters: paths to discovery and overview. Physiology and pathology of chloride transporters and channels in the nervous system: from molecules to disease.

[29] Gibson JS, Al Balushi HWM, Hannemann A, Rees D (2015). Sickle cell disease and 5HMF: the search for effective treatments. Drugs Future.

[30] Hall AC, Ellory JC (1986). Evidence for the presence of volume-sensitive KCl transport in 'young' human red cells. Biochim Biophys Acta.

[31] Hannemann A, Flatman PW (2011). Phosphorylation and transport in the Na-K-2Cl cotransporters, NKCC1 and NKCC2A, compared in HEK-293 cells. PLoS ONE.

[32] Hoffman EK, Sjoholm C, Simonsen LO (1981). Anion-cation co-transport and volume regulation in Ehrlich ascites tumour cells. J Physiol.

[33] Jennings ML, Al-Rohil N (1990). Kinetics of activation and inactivation of swelling-stimulated K/Cl transport: the volume-sensitive parameter is the rate constant for inactivation. J Gen Physiol.

[34] Jennings ML, Schulz RK (1991). Okadaic acid inhibition of KCl cotransport: evidence that protein dephosphorylation is necessary for activation of transport by either swelling or N-ethylmaleimide. J Gen Physiol.

[35] Joiner CH, Rettig RK, Jiang M, Franco RS (2004). KCl cotransport mediates abnormal sulfhydryl-dependent volume regulation in sickle erythrocytes. Blood.

[36] Kahle KT, Rinehart J, Ring A, Gimenez I, Gamba G, Hebert SC, Lifton RP (2006). WNK protein kinases modulate cellular Cl^-^ flux by altering the phosphorylation state of the Na-K-Cl and K-Cl cotransporters. Physiology.

[37] Kaji DM, Gasson C (1995). Urea activation of K-Cl cotransport in human erythrocytes. Am J Physiol.

[38] Lauf PK, Theg BE (1980). A chloride dependent K^+^ flux induced by *N*-ethylmaleimide in genetically low K^+^ sheep and goat erythrocytes. Biochem Biophys Res Commun.

[39] Lauf PK, Bauer J, Adragna NC, Fujise H, Martin A, Zade-Oppen M, Ryu KH, Delpire E (1992). Erythrocyte K-Cl cotransport: properties and regulation. Am J Physiol.

[40] Low PS, Rathinavelu P, Harrison ML (1993). Regulation of glycolysis via reversible enzyme binding to the membrane protein, band 3. J Biol Chem.

[41] Merciris P, Hardy-Dessources MD, Giraud F (2001). Deoxygenation of sickle cells stimulates Syk tyrosine kinase and inhibits a membrane tyrosine phosphatase. Blood.

[42] Merciris P, Claussen WJ, Joiner CH, Giraud F (2003). Regulation of K-Cl cotransport by Syk and Src protein tyrosine kinases in deoxygenated sickle cells. Pflugers Arch - Eur J Physiol.

[43] Motais R, Garcia-Romeu F, Borgese F (1987). The control of Na/H exchange by molecular oxygen in trout erythrocytes. J Gen Physiol.

[44] Muzyamba MC, Cossins AR, Gibson JS (1999). Regulation of Na^+^-K^+^-2Cl^-^ cotransport in turkey red cells: the role of oxygen tension and protein phosphorylation. J Physiol.

[45] Pan D, Kalfa TA, Wang D, Risinger M, Crable S, Ottlinger A, Chandra S, Mount DB, Hubner CA, Franco RS, Joiner CH (2011). K-Cl cotransporter gene expression during human and murine erythroid differentiation. J Biol Chem.

[46] Pellegrino CM, Rybicki AC, Musto S, Nagel RL, Schwartz RS (1998). Molecular identification of erythroid K:Cl cotransporter in human and mouse erythroleukemic cells. Blood Cell Mol Dis.

[47] Piechotta K, Lu J, Delpire E (2002). Cation chloride cotransporters interact with the stress-related kinases Ste20-related proline-alanine-rich kinase (SPAK) and oxidative stress response 1 (OSR1). J Biol Chem.

[48] Rees DC, Williams TN, Gladwin MT (2010). Sickle-cell disease. Lancet.

[49] Richardson C, Alessi DR (2008). The regulation of salt transport and blood pressure by the WNK-SPAK/ORS1 signalling pathway. J Cell Sci.

[50] Rinehart J, Maksimova YD, Tanis JE, Stone KL, Hodson CA, Zhang J, Risinger M, Pan W, Wu D, Colangelo CM, Forbush B, Joiner CH, Gulcicek EE, Gallagher PG, Lifton RP (2009). Sites of regulated phosphorylation that control K-Cl cotransporter activity. Cell.

[51] Rust MB, Alper SL, Rudhard Y, Shmukler BE, Vicente R, Brugnara C, Trudel M, Jentsch TJ, Hubner CA (2007). Disruption of erythroid K-Cl cotransporters alters erythrocyte volume and partially rescues erythrocyte dehydration in SAD mice. J Clin Investig.

[52] Starke LC, Jennings ML (1993). KCl cotransport in rabbit red cells: further evidence for regulation by protein phosphatase type I. Am J Physiol.

[53] Steinberg MH (1999). Management of sickle cell disease. N Engl J Med.

[54] Weaver YR, Cossins AR (1995). Protein tyrosine phosphorylation regulates the KCl cotransporter in trout red cells. J Physiol.

[55] Wesseling MC, Wagner-Britz L, Nguyen DB, Asanidze S, Mutua J, Mohamed N, Hanf B, Ghashghaeinia M, Kaestner L, Bernhardt I (2016). Novel insights into the regulation of phosphatidylserine exposure in human red blood cells. Cell Physiol Biochem.

[56] Wilson FH, Disse-Nicodeme S, Choate KA, Ishikawa K, Nelson-Williams C, Desitter I, Gunel M, Milford DV, Lipkin GW, Achard JM, Feely MP, Dussol B, Berland Y, Unwin RJ, Mayan H, Simon DB, Farfel Z, Jeunemaitre X, Lifton RP (2001). Human hypertension caused by mutations in WNK kinases. Science.

[57] Xu JC, Lytle C, Zhu TT, Payne JA, Benz EJ, Forbush BI (1994). Molecular cloning and functional expression of the bumetanide-sensitive Na-K-Cl cotransporter. Proc Natl Acad Sci U S A.

[58] Xu B, English JM, Wilsbacher JL, Stippec S, Goldsmith EJ, Cobb MH (2000). WNK1, a novel mammalian serine/threonine protein kinase lacking the catalytic lysine in subdomain II. J Biol Chem.

[59] Yagi YI, Avbe K, Ikebukuro K, Sode K (2009). Kinetic mechanism and inhibitor characterization of WNK1 kinase. Biochemistry.

[60] Yamada K, Park HM, Rigel DF, DiPetrillo K, Whalen EJ, Anisowicz A, Beil M, Berstler J, Brocklehurst CE, Burdick DA, Caplan SL, Capparelli MP, Chen G, Chen W, Dale B, Deng L, Fu F, Hamamatsu N, Harasaki K, Herr T, Hoffmann P, Hu QY, Huang WJ, Idamakanti N, Imase H, Iwaki Y, Jain M, Jeyaseelan J, Kato M, Kaushik VK, Kohls D, Kunjathoor V, LaSala D, Lee J, Liu J, Luo Y, Ma F, Mo R, Mowbray S, Mogi M, Ossola F, Pandey P, Patel SJ, Raghavan S, Salem B, Shanado YH, Trakshel GM, Turner G, Wakai H, Wang C, Weldon S, Wielicki JB, Xie X, Xu L, Yagi YI, Yasoshima K, Yin J, Yowe D, Zhang JH, Zheng G, Monovich L (2016). Small-molecule WNK inhibition regulates cardiovascular and renal function. Nat Chem Biol.

[61] Zagorska A, Pozo-Guisado E, Boudeau J, Vitari AC, Rafiqi FH, Thastrup J, Dean M, Campbell DG, Morrice NA, Prescott AR, Alessi DR (2007). Regulation of activity and localization of the WNK1 protein kinase by hyperosmotic stress. J Cell Biol.

[62] Zhang J, Gao G, Begum G, Wang J, Khanna AR, Shmukler BE, Daubner GM, de los Heros P, Davies P, Varghese J, Bhuiyan MI, Duan D, Alper SL, Sun D, Elledge SJ, Alessi DR, Kahle KT (2016). Functional kinomics establishes a critical mode of volume-sensitive cation-Cl^-^ cotransporter regulation in the mammalian brain. Sci Rep.

[63] Zheng S, Krump NA, McKenna MM, Li Y-H, Hannemann A, Garrett LJ, Gibson JS, Bodine DM, Low PS (2019). Regulation of erythrocyte Na^+^K^+^2Cl^-^ cotransport by an oxygen-switched kinase cascade. J Biol Chem.

